# A machine learning-based service for estimating quality of genomes using PATRIC

**DOI:** 10.1186/s12859-019-3068-y

**Published:** 2019-10-03

**Authors:** Bruce Parrello, Rory Butler, Philippe Chlenski, Robert Olson, Jamie Overbeek, Gordon D. Pusch, Veronika Vonstein, Ross Overbeek

**Affiliations:** 1 Fellowship for Interpretation of Genomes, Burr Ridge, 60527 IL USA; 2 University of Chicago, Chicago, 60637 IL USA; 3 Computing, Environment, and Life Sciences Directorate, Argonne National Laboratory, 4200 S. Cass Avenue, Lemont, 60439 IL USA

**Keywords:** CheckM, RAST, Genome annotation, Random forest, Machine learning, Metagenomics, Genome quality, Supervised learning

## Abstract

**Background:**

Recent advances in high-volume sequencing technology and mining of genomes from metagenomic samples call for rapid and reliable genome quality evaluation. The current release of the PATRIC database contains over 220,000 genomes, and current metagenomic technology supports assemblies of many draft-quality genomes from a single sample, most of which will be novel.

**Description:**

We have added two quality assessment tools to the PATRIC annotation pipeline. EvalCon uses supervised machine learning to calculate an annotation consistency score. EvalG implements a variant of the CheckM algorithm to estimate contamination and completeness of an annotated genome.We report on the performance of these tools and the potential utility of the consistency score. Additionally, we provide contamination, completeness, and consistency measures for all genomes in PATRIC and in a recent set of metagenomic assemblies.

**Conclusion:**

EvalG and EvalCon facilitate the rapid quality control and exploration of PATRIC-annotated draft genomes.

## Background

The Pathosystems Resource Integration Center (PATRIC) [1, 2] currently contains over 220,000 genomes, some of which come from metagenomic samples. The field of metagenomics has recently seen increases in the quality and quantity of genomes that can be assembled from a sample, and the bulk of future genomes added to PATRIC will likely come from metagenomes. A recent metagenomic analysis by Pasolli et al. has produced 150,000 draft genomes [3] that are being considered for inclusion in the PATRIC database. Such evaluations and metagenomic assembly methods themselves depend on rapid and reliable draft genome quality assessment.

Current methods for automated evaluation of draft genomes rely on scores computed from the absence or overabundance of lineage-specific marker genes. Anvi’o estimates completion and redundancy based on Hidden Markov Model-derived profiles of expected single-copy genes in a lineage [4]. BUSCO uses evolutionarily-informed expectations of gene content in near-universal, single-copy genes pulled from OrthoDB v9 to calculate the completeness of draft genomes [5]. CheckM, which uses collocated single-copy, ubiquitous, lineage-specific genes to estimate measures of completeness and contamination [6], has been used to compare the effectiveness of assembly methods [7] and to evaluate the quality of metagenomic draft genomes [3].

In this paper, in addition to completeness and contamination, we introduce *consistency,* a complementary metric of genome quality applicable to RAST-annotated genomes [8]. The RAST system annotates genomes using a controlled vocabulary derived from a set of manually curated gene subsystems [9].

## Construction

### Consistency

We wish to define a measure of *annotation self-consistency* as an extension of the notions of completeness and contamination. We must first define some terms: A genome contains a set of *protein encoding genes* (PEGs). Each PEG encodes a single protein implementing a *function*, which consists of one or more *roles*. A set of roles that are related in some defined way constitutes a *subsystem.* The notion of subsystem generalizes and abstracts the notion of a biochemical pathway to include any biologically relevant set, such as a structural complex or a subnetwork.

**Fig. 1:**
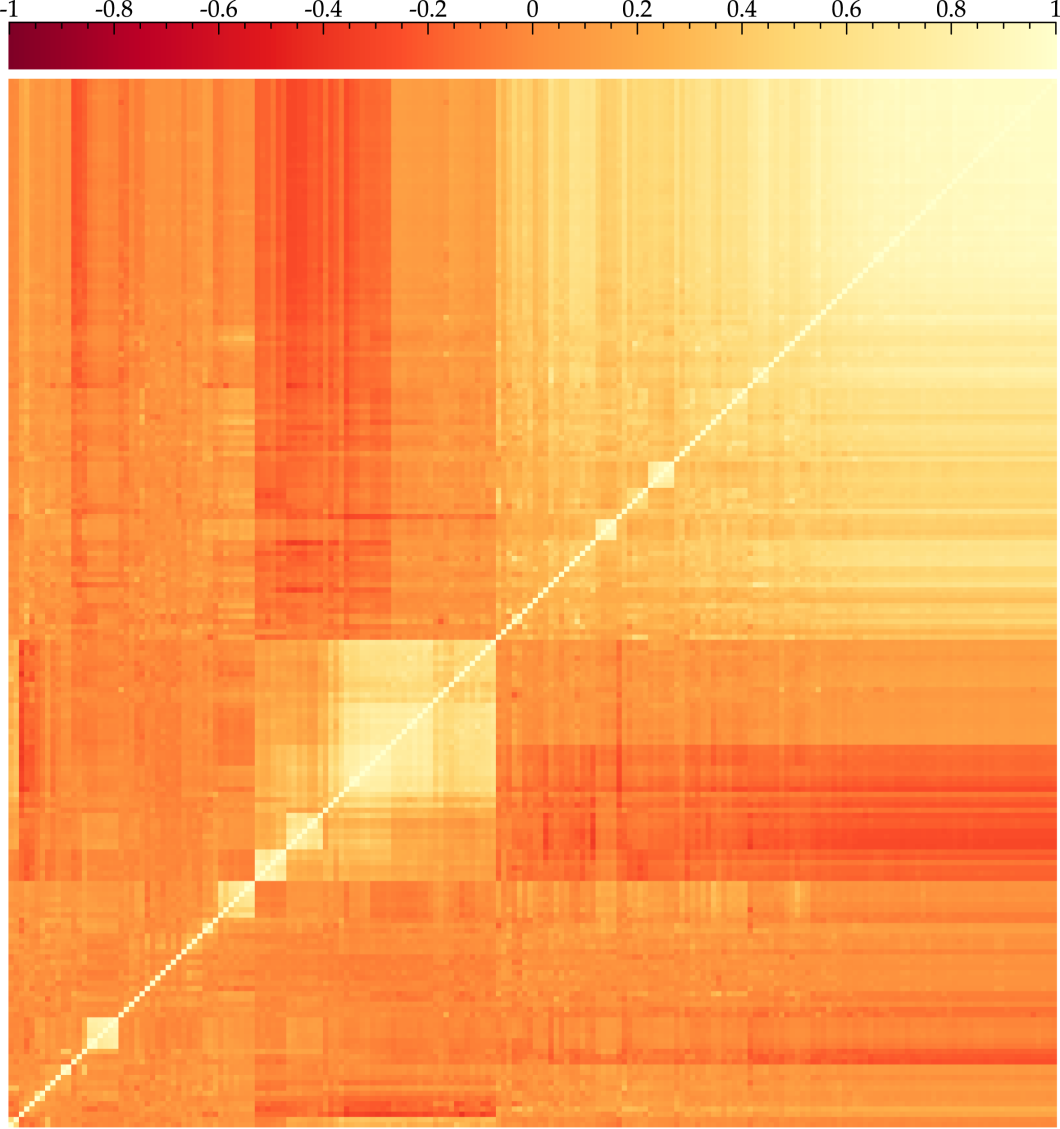
Role correlations. Heatmap of role-role correlations for a subset of roles clustered according to the dendrogram clustering method in R. Roles are arranged according to their positions in a dendrogram (not shown) computed according to their mutual correlations. In particular, roles that are clustered together in the dendrogram will appear close to one another in the diagram; borders with high contrast correspond to divisions between higher-order clusters. This algorithm maximizes contrast in the heatmap at such boundaries and results in light-colored blocks of strongly correlated roles. High correlations along the diagonal correspond to highly conserved small sets of roles, e.g. subunits of a single protein complex, and all roles are fully correlated with themselves (*ρ*=1). While it is apparent from visual inspection of the blocks in the heatmap that there is an underlying structure to these role-role correlations, the actual nature of this structure can be nonapparent and difficult to characterize precisely. EvalCon uses machine learning to learn these structures from role-role correlations, thereby eliminating the need for an a priori characterization

The *multiplicity* of a role refers to the number of PEGs implementing that role in a given genome. Because individual genes do not function in isolation but work together to build structures and perform functions within a genome, we observe correlated patterns of role multiplicities, many but not all of which correspond to our manually curated subsystems. Figure 1, which shows a heatmap of role-to-role correlations for a subset of roles, illustrates the kind of patterns we seek to predict.

In most cases the multiplicity for a set of correlated roles will be either one or zero (all present with a single copy, or all absent); however in some cases the multiplicities may be higher, because of gene duplications or multiple copies of an operon or the presence of *mobile elements* such as transposons, phage insertions, or plasmids.

Since we do not yet have a complete manual characterization of all role correlations, we shall use *machine learning* to capture the most significant of these correlations.

We shall call a role *strongly predictable* by some predictor if, under 5-fold cross-validation, its multiplicity can be predicted with better than 93% accuracy as estimated by Tukey’s trimean [10, p. 3069] and less than 5% accuracy dispersion as estimated by the *interquartile range* (IQR) [10, p. 1505]; we chose these two measures because they are robust against outliers. The set of strongly predictable roles depends on the predictor being used. Using only the set of strongly predictable roles for consistency checking reduces the probability of obtaining false positive and false negative inconsistencies between observed and predicted role multiplicities.

We define consistency for a genome and role multiplicity predictor as the percentage of agreement between the annotated and predicted role multiplicities. We define *fine consistency score* to be the percentage of strongly predictable roles whose annotated multiplicity matches their predicted multiplicity exactly, and we define *coarse consistency score* to be the percentage of roles whose annotated occurrence or nonoccurrence matches their predictor.

### EvalCon

**Fig. 2:**
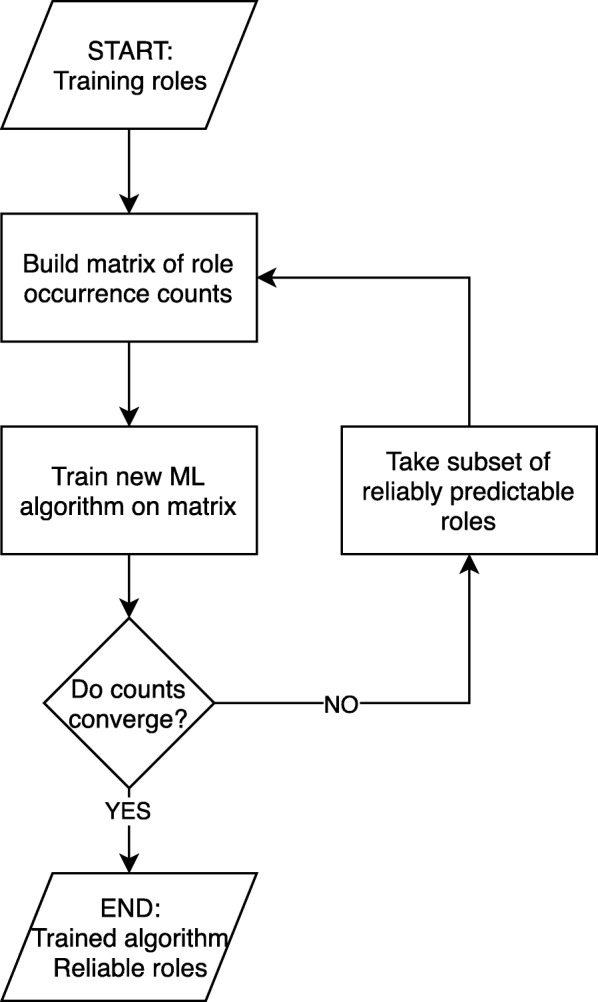
Map of the process of training EvalCon given a machine learning algorithm and a set of training roles. For the development of EvalCon in PATRIC, the training roles were kept constant, and a variety of machine learning predictors were tested with this process

Given a RAST-annotated genome and a machine learning algorithm trained on a set of reliably predictable roles, EvalCon implements a jackknife predictor of role multiplicity and returns a vector of predicted multiplicities for each role in the genome of interest (Fig. 2).

For our training data, we used a set of Additional file 1 that have been manually curated by the SEED project [11], We selected from these genomes Additional file 2 that are: (1) members of subsystems (and may therefore be expected to be highly correlated with each other), (2) appear in at least 10% of the training genomes, and (3) have multiplicities of no more than 5 (thereby excluding roles within mobile elements and other genomic regions that have highly variable counts).

We then iteratively (1) built Additional file 3, (2) trained a machine learning algorithm to predict the count of each role for a genome based on the counts of all other roles, (3) selected Additional file 4, and (4) used this set of *reliably predictable* roles to build a matrix for the next iteration and Additional file 5.

To date we have built predictors using a number of classical machine-learning methods and one deep learning method. We chose these models for their ability to work with discrete ordered data and to model complex decision processes.

We used the Python *scikit-learn* package to construct the following classical predictors: linear discriminant analysis, logistic regression, three random forest-based models (random forest regressor, random forest classifier, and ExtraTrees), and XGBoost. The input matrix for these predictors was formed from the role multiplicities of all roles except the target role, which was used as the output label. For regression-based models, we rounded the output to integer values at the end. We evaluated the training time, size of the final set of reliably predictable roles, and the average accuracy of each model’s predictions.

We used the Python *Keras* 2.0.2 API ordinary deep neural network with the Sequential model type following a standard classifier structure. The role multiplicities formed the input layer (one neuron per role) followed by one fully connected hidden layer of 16 nodes using the ReLU (Rectified Linear Unit) activation function. The output layer had six nodes, one for each possible multiplicity level the target role could have, and used the softmax activation function. The network was compiled with a learning rate of 0.001 in the Adam optimizer and the sparse_categorical_crossentropy loss function. The sparse version of the loss function allows the label matrix to be formatted as integers, converted internally by Keras into the one-hot array that the network is trained to produce. After one training session the networks were able to reliably predict a set of 1010 roles. Using the same iterative process as performed on the scikit-learn predictors, the networks converged on a set of 812 reliable roles.

The performance of the machine learning algorithms tested was measured on two criteria: the number of roles that exceeded the 93% accuracy threshhold in the first iteration of role selection and the time required for training of the algorithm. All predictors were built by using 32 processes run in parallel using the scikit-learn module joblib. The results are summarized in Table 1.

**Table 1:** Summary of machine learning algorithm performance

Algorithm	Parameters	Training Time (s/role)	Roles >93% Accuracy	Avg. Accuracy (%)
Linear Discriminant Analysis	Default	3.51	785	87.4
Logistic Regression	Optimized	9.16	1081	91.4
Random Forest Regressor	Optimized	1.35	1299	92.7
ExtraTrees Classifier	Optimized	0.89	1405	93.4
XGBoost	Optimized	7.40	1417	93.6
Random Forest Classifier	Optimized	1.01	1423	93.5

Of all the predictors tested, the random forest classifier produced 1423 reliably predictable roles after the first iteration, outperforming all other predictors. At a training time of 1.01 s per role, it is also the second-fastest predictor (after the ExtraTrees classifier). We therefore selected the random forest classifier to be the predictor for EvalCon based on these criteria, and iterated the training and role-selection to convergence as described above, yielding a final set of 1353 reliably predictable roles. (These data may be found in the electronic supplement.)

### EvalG

EvalG implements a variant of the basic CheckM algorithm using direct calls into the PATRIC database and user workspaces. For faster performance, it omits the gene-calling and BLAST phase of the full CheckM algorithm and uses RAST annotations instead.

We also use our own set of marker genes derived from PATRIC genomes; the presence or absence of these marker genes is reported as *universal roles* in the problematic roles report. Based on annotated genome data in PATRIC, we generated sets of marker roles for multiple taxonomic groupings representing species, genus, and family-level similarity. For a given taxonomic grouping, a marker role is one that occurs exactly once in 97% or more of the genomes in that grouping. The marker roles were then clustered based on whether they co-occurred in 90% or more members of a given taxonomic grouping.

For computing the completeness and contamination scores, each marker role is given a weight of $\frac {1}{N}$, where *N* represents the size of the clustered set. For a genome being evaluated, we find the most granular taxonomic grouping containing the incoming genome and then run through all the features implementing that group’s set of marker roles in the draft genome.

Designating as *M* the set of marker roles, as *O* the set of roles that occur, as *n*_*x*_ the number of occurrences of a role *x*∈*M*∪*O*, and *N*_*x*_ as the size of the clustered set to which *x* belongs, EvalG computes the contamination and completeness scores as follows.

1 $$\begin{array}{*{20}l} \text{Contamination} =& \frac{\sum_{x\in O}(n_{x}-1)/N_{x}}{\sum_{x \in O} n_{x}/N_{x}} \end{array} $$

2 $$\begin{array}{*{20}l} \text{Completeness} =& \frac{\sum_{x \in O} 1/N_{x}}{\sum_{x \in M} 1/N_{x}} \end{array} $$

This definition of contamination differs from the value calculated by CheckM to produce a value in the 0–100 range. In response to PATRIC user preferences, this latter value corresponds to the more intuitive notion of contamination as the percentage of the draft genome that can be attributed to contamination.

## Utility

### Integration into the annotation pipeline

Quality reporting is an automatic part of PATRIC’s annotation service [12], comprehensive genome analysis pipeline [13], and metagenomic binning service [14]. Because we use lineage-specific marker genes, computing an accurate estimate of a genome’s completeness and consistency depends on accurately knowing that genome’s taxonomic group. A genome’s taxonomy ID is input by the user of the annotation service and the comprehensive genome analysis pipeline, whereas it is estimated automatically for each putative genome within the metagenome binning service.

Each completed PATRIC annotation job creates a directory containing an annotated genome as well as detailed EvalG and EvalCon quality reports for that genome. These reports include the completeness and contamination, the fine and coarse consistency scores, the counts for predicted roles, overrepresented and underrepresented roles, and a detailed structured-language table of potentially problematic roles with links to related features. These reports, including the role multiplicities predicted by the EvalCon predictor, are automatically made available in JSON, structured plaintext, and structured HTML formats.

EvalCon and EvalG rely on RAST annotations and lack a gene-calling step of their own. This design makes them much faster but also inseparable from the rest of the annotation pipeline. As the quality of annotations improves, the completeness, contamination, and consistency scores should become more reliable; the completeness score in particular should approach the CheckM completeness score.

### Problematic roles report

A *problematic roles report* is found at the end of the genome quality report. It contains the following columns: (1) role, (2) predicted count, (3) annotated count, (4) feature link, and (5) comment. The feature link allows a user to view all of the features implementing the role of interest; if no such features are found, no link is given. The comment field contains automatically generated structured text that is meant to help the user determine why a particular role may be problematic.

*Universal roles* are roles that EvalG expects to occur exactly once for a given taxonomic grouping. The absence of a universal role in a genome lowers the completeness score, and redundancies in universal roles increase the contamination score. In the problematic roles report table, the comment field for each problematic universal role will include the phrase “universal role,” which helps users understand the EvalG scores in more detail.

The contig on which it is found and link to the *Compare Region Viewer* [15], a PATRIC tool that allows users to see the feature in its immediate context on the chromosome alongside its closest relatives in the contexts of their respective genomes. Features that are short, appear on short contigs, or are located close to the edge of a contig are marked accordingly in the comment field.

**Fig. 3:**
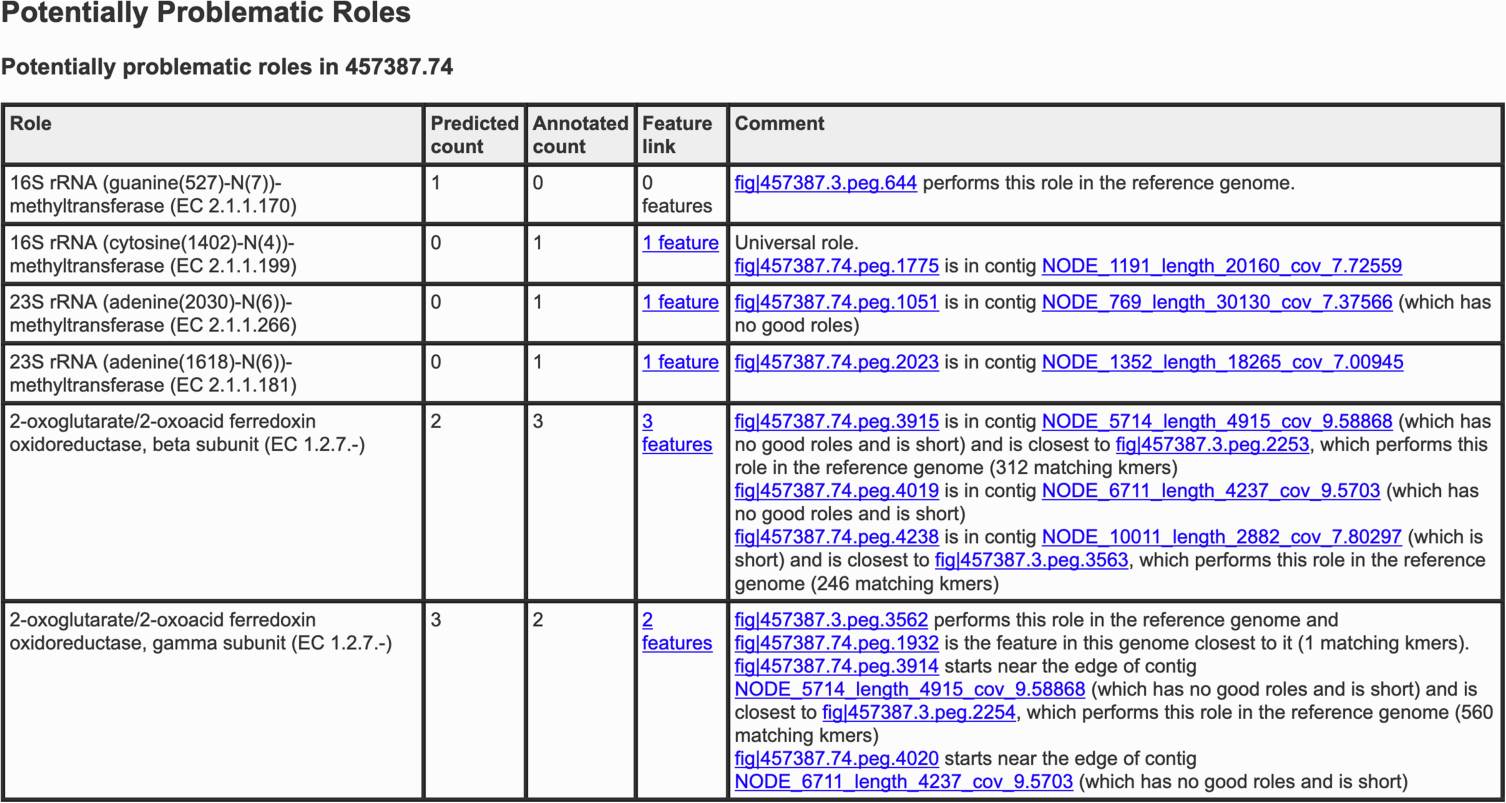
Sample problematic roles report. First six rows of a problematic roles report for a draft genome produced by the PATRIC metagenome binning service. The first four rows represent coarse inconsistencies: one role that is predicted but is not observed, and three roles which are observed but not predicted. The fifth row represents a fine inconsistency corresponding to an extra PEG, and the sixth represents a fine inconsistency corresponding to a missing PEG. Where applicable, the comment field notes universal roles, contig membership for observed roles, short contigs, contigs with no good roles, features appearing near the ends of contigs, and closest features on the reference genome

An excerpt from a problematic roles report is provided in Fig. 3, which displays examples of both coarse inconsistencies (missing and unanticipated roles) and fine inconsistencies (too many or too few features implementing a role). For each problematic role the comments will contain a link to the relevant contig; the report also notes contigs that are short or contain no reliably predictable roles. For any universal role, the comments begin with the phrase “Universal role.” All references to PEGs link to the Compare Region Viewer tool.

Problematic roles appearing fewer times than predicted (possibly not at all) will contain links to PEGs implementing the corresponding role in the reference genome where possible. If no PEG for a predicted role exists, its absence may often be traced back to a frameshift error or truncation by contig boundary that prevented the PEG from being called. On occasion, however, the missing role represents a predictor error instead of a problem with the genome or its annotation.

For problematic roles that occur more frequently than predicted, the comment field will include links to the contigs for each PEG. The PEGs most likely to be problematic are those that are not close to PEGs implementing related roles within the reference genome (and may therefore represent an instance of contamination) or that have been fragmented by a frameshift or assembly error. On occasion, however, an overrepresented role may be the result of a genuine mutation or gene duplication.

As yet no automatic criterion can distinguish between genuine changes in a genome versus sequencing, assembly, or annotation errors. The expert user must determine by inspection what has mostly likely happened.

## Discussion

### EvalCon performance

We have introduced a measure of the quality of annotation consistency, the *fine consistency score*, which is the percentage of functional roles with a predicted multiplicity matching the RAST-annotated multiplicity. We verified the accuracy of fine consistency scores and measured the effects of genome incompleteness and contamination by training a separate set of random forest predictors on 80% of the original training data and running validation tests on the other 20%. We examined role count predictions for both the unmanipulated validation data and genomes with simulated contamination and incompleteness.

**Fig. 4:**
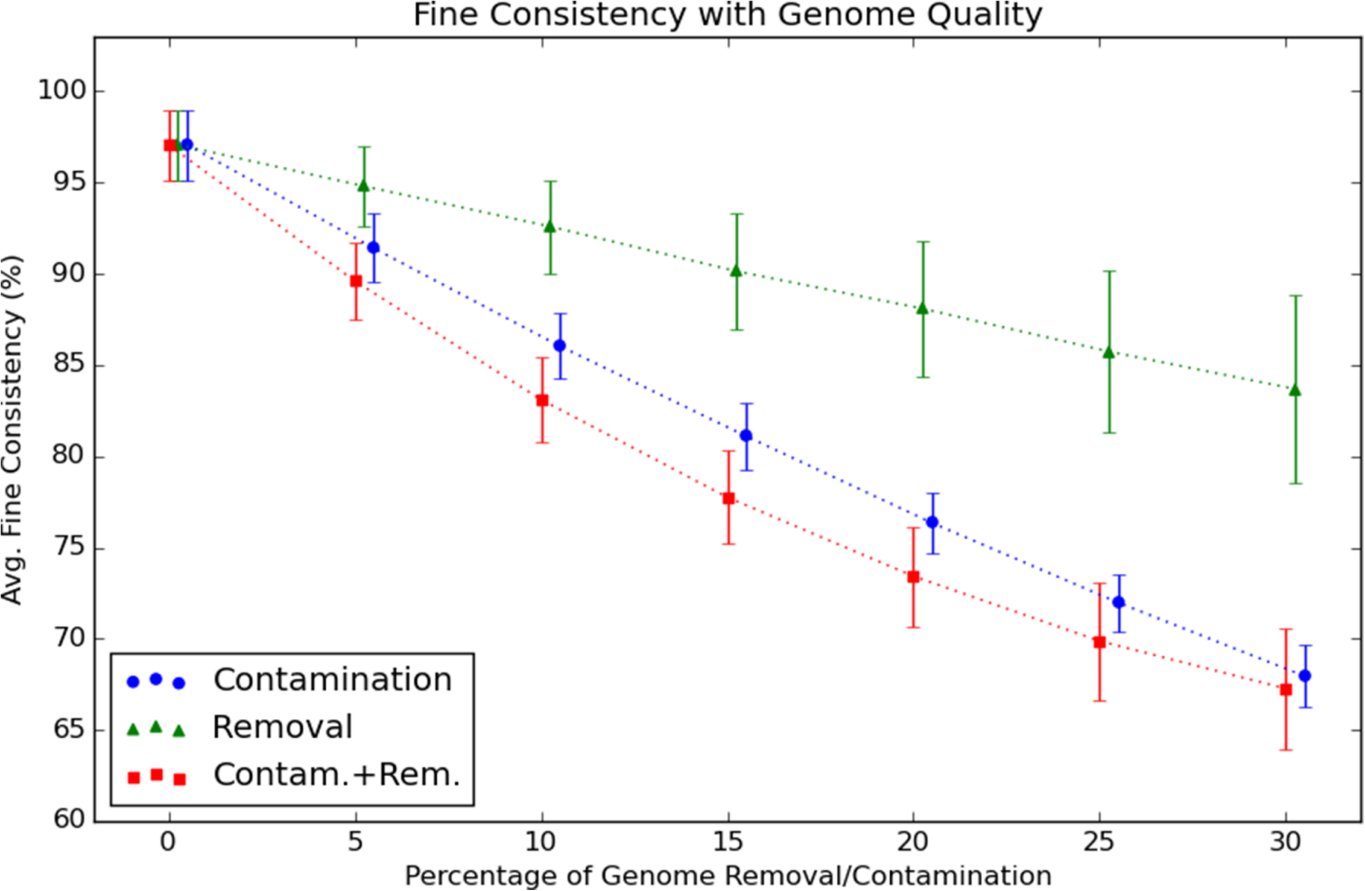
Fine consistency as a function of quality. Average fine consistency scores for 193 validation genomes under conditions of simulated incompleteness and contamination

**Fig. 5:**
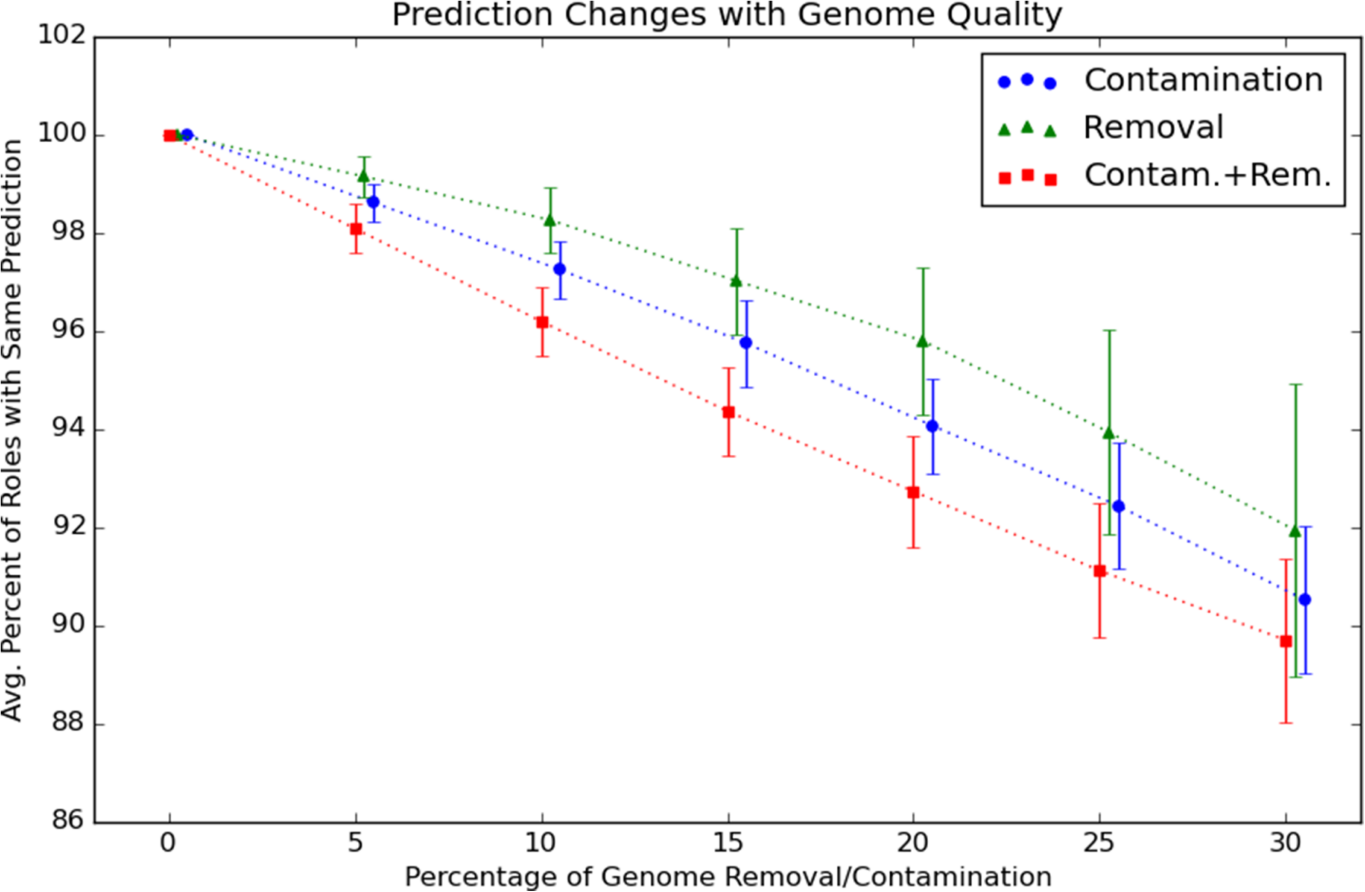
Changes in predictor as a function of quality. Average percentage of predictions remaining constant for 193 validation genomes under conditions of simulated incompleteness and contamination

Genome incompleteness was simulated by lowering a percentage of randomly selected role counts by one (if the role has a count greater than zero); contamination was simulated by increasing a percentage of randomly selected role counts with replacement. The results are shown in Figs. 4 and 5. The role predictors generally performed well on novel data, even after training only on 80% of the available genomes. With no induced contamination or incompleteness, the 193 validation genomes had an average fine consistency score of 97 ±2*%*. Figure 4 shows average fine consistency scores (with standard deviations as error bars) for validation genomes, given a certain percentage of contamination, incompleteness, or both. As expected, fine consistency scores decrease approximately linearly with increasing contamination and incompleteness levels. The decrease with percentage contamination is approximately 1:1, but the decrease with incompleteness is more gradual because many genomes have a substantial fraction of role counts equal to zero.

Figure 5 shows the percentage of unchanged role predictions with increasing levels of genome contamination and incompleteness. A set of role predictors used to evaluate genome quality should ideally predict the same role counts even with substantial genome contamination and incompleteness; and we see that even at 30% incompleteness or contamination, for most genomes less than 10% of role count predictions are altered.

Average fine consistency scores for genomes with both artificial contamination and incompleteness decrease linearly to ∼20% and then begin to level off.

### EvalG performance

The completeness scores provided by EvalG differed from CheckM values by a mean of 5.1% and a median of 3.2%. The contamination scores provided by EvalG are calculated by using a different definition from that used by CheckM and therefore are not immediately comparable; EvalG calculates contamination over all *counts of* universal roles, whereas CheckM calculates contamination over the *number* of universal roles appearing in the sample. Thus, a highly diverse metagenomic sample may yield a CheckM contamination score over 100%, whereas the EvalG contamination score tends asymptotically to 100%.

EvalG is substantially faster (and therefore more scalable within the PATRIC environment) than CheckM. On a random sample of 1000 genomes in PATRIC, EvalG took 4 milliseconds per genome on its own, and the combined EvalG and EvalCon analysis took an average of 1.51 s per genome. CheckM runtime is on the order of several minutes on the same hardware. Quality scores are recalculated for all genomes in PATRIC on a quarterly basis, so this represents a substantial reduction in computational demands at scale. This reduction in time is driven by the use of the RAST-annotated features (which are already an integral part of the PATRIC framework), rather than running PRODIGAL and BLAST de novo, which together take an average of 5 min.

### Analysis

We have run the EvalG and EvalCon algorithms on the Additional file 6 and Additional file 7 and have tabulated the results. Our analysis verifies the quality of the Pasolli et al. metagenomic assemblies [3]: we identified 76,402 genomes meeting the PATRIC criteria of high quality (contamination ≤10%, consistency ≥87%, completeness ≥80%), which is close to the Pasolli et al. figure of 70,178 high-quality genomes. These genomes will be added to the PATRIC database in the near future. (These quality reports may be found in the electronic supplement.)

The EvalG estimates for completeness differed from CheckM by an average of 6.5% and a median of 3.3%. Since the Pasolli et al. estimate uses CheckM and does not account for consistency, this suggests that EvalG is a viable quality checker even for data representing uncharacterized or undersampled taxonomies. The availability of more high-quality annotated reference genomes should further improve the performance of EvalG and EvalCon.

### Future directions

The notion of a strongly predictable role, which is dependent on the machine learning predictor being used, is currently used only as a heuristic to find roles whose multiplicities behave in a predictable way. This set can also be said to correspond to the subset of roles that, across all organisms, exhibit an abstract notion of structure. Further exploration of this set of and corresponding expression data with machine learning may be a viable first step toward a machine learning-based characterization of the structure of unicellular life.

## Conclusions

We have presented a new service that provides rapid estimates of completeness, contamination, and annotation self-consistency for RASTtk-annotated genomes. It additionally flags potentially problematic gene calls and annotations. These tools can be accessed through the PATRIC annotation service.

The submitted manuscript has been created by UChicago Argonne, LLC, Operator of Argonne National Laboratory (“Argonne”). Argonne, a U.S. Department of Energy Office of Science laboratory, is operated under Contract No. DE-AC02-06CH11357. The U.S. Government retains for itself, and others acting on its behalf, a paid-up nonexclusive, irrevocable worldwide license in said article to reproduce, prepare derivative works, distribute copies to the public, and perform publicly and display publicly, by or on behalf of the Government. The Department of Energy will provide public access to these results of federally sponsored research in accordance with the DOE Public Access Plan. http://energy.gov/downloads/doe-public-access-plan.

## Supplementary information

**Additional file 1** EvalCon genome names. This file contains (row index, genome name) tuples for both the training data matrix and the converged matrix.

**Additional file 2** EvalCon training role names. This file contains (column index, role name) tuples for the training data matrix.

**Additional file 3** EvalCon training multiplicity matrix. This is the multiplicity matrix used to train the machine learning predictors for EvalCon. Each line of this file represents a single genome, with tab-separated multiplicities for each role. Its rows are labeled in genome_names.txt and its columns are labeled in all_roles.txt.

**Additional file 4** EvalCon reliable role names. This file contains (column index, role name) tuples for the converged matrix. All indices start at zero. This is the subset of roles in all_roles.txt found to be reliably predictable under the random forest predictor.

**Additional file 5** EvalCon converged multiplicity matrix. This is the multiplicity matrix with its columns pared down to the set of reliably predictable roles. Each line of this file represents a single genome, with tab-separated multiplicities for each role. Its rows are labeled in genome_names.txt and its columns are labeled in reliable_roles.txt.

**Additional file 6** PATRIC quality report. This is a report of EvalCon and EvalG scores for all public genomes in PATRIC. Columns, in order, are: PATRIC genome ID, genome name, EvalCon fine consistency score, EvalG completeness score, EvalG contamination score, “Good,” and “Good Seed.” Genomes marked “good” meet the following criteria: (1) contamination ≤10%, (2) fine consistency ≥87%, and (3) completeness ≥80%. Genomes marked “good seed” have a single copy of the phenylalanine tRNA synthetase, alpha subunit (pheS) gene of appropriate length (209–405 amino acid residues for bacteria, 293-652 for archaea).

**Additional file 7** Pasolli et al. quality report. This is a report of EvalCon and EvalG scores for high- and medium-quality genomes assembled in the Pasolli et al. study. Columns, in order, are Pasolli et al. genome name, CheckM completeness score, CheckM contamination score, PATRIC genome ID, Scientific name of organism, EvalG completeness score, EvalG contamination score, EvalCon coarse consistency score, EvalCon fine consistency score, and “good?” Genomes marked “good” in this table will meet the following criteria: (1) contamination ≤10%, (2) fine consistency ≥87%, and (3) completeness ≥80%, and (4) a single copy of pheS of appropriate length.

## Data Availability

EvalCon and EvalG are available as part of the PATRIC annotation service, which can be accessed at https://patricbrc.org/app/Annotation. The training data for EvalCon and quality reports for PATRIC and Pasolli et al. genomes are available in the additional files section of this paper. The Pasolli et al. dataset is available at http://segatalab.cibio.unitn.it/data/Pasolli_et_al.html.

## Abbreviations

IQR: Interquartile range
PATRIC: Pathosystems Resource Integration Center
PEG: Protein encoding gene
ReLU: Rectified linear unit

## References

[1] Wattam AR, Davis JJ, Assaf R, Boisvert S, Brettin T, Bun C, Conrad N, Dietrich EM, Disz T, Gabbard J, Gerdes S, Henry CS, Kenyon R, Machi D, Mao C, Nordberg EK, Olsen G, Murphy-Olson DE, Olson R, Overbeek R, Parrello B, Pusch GD, Shukla M, Vonstein V, Warren A, Xia F, Yoo H, Stevens R (2017). Improvements to patric, the all-bacterial bioinformatics database and analysis resource center. Nucleic Acids Res.

[2] Snyder EE, Kampanya N, Lu J, Nordberg EK, Karur HR, Shukla M, Soneja J, Tian Y, Xue T, Yoo H, Zhang F, Dharmanolla C, Dongre NV, Gillespie JJ, Hamelius J, Hance M, Huntington KI, Jukneliene D, Koziski J, Mackasmiel L, Mane SP, Nguyen V, Purkayastha A, Shallom J, Yu G, Guo Y, Gabbard J, Hix D, Azad AF, Baker SC, Boyle SM, Khudyakov Y, Meng XJ, Rupprecht C, Vinje J, Crasta OR, Czar MJ, Dickerman A, Eckart JD, Kenyon R, Will R, Setubal JC, Sobral BWS (2007). Patric: the vbi pathosystems resource integration center. Nucleic Acids Res.

[3] Pasolli E, Asnicar F, Manara S, Zolfo M, Karcher N, Armanini F, Beghini F, Manghi P, Tett A, Ghensi P, Collado MC, Rice BL, DuLong C, Morgan XC, Golden CD, Quince C, Huttenhower C, Segata N (2019). Extensive unexplored human microbiome diversity revealed by over 150,000 genomes from metagenomes spanning age, geography, and lifestyle. Cell.

[4] Eren AM, Esen ÖC, Quince C, Vineis JH, Morrison HG, Sogin ML, Delmont TO, van Gulik W (2015). Anvi’o: an advanced analysis and visualization platform for ‘omics data. PeerJ.

[5] Kriventseva EV, Zdobnov EM, Simão FA, Ioannidis P, Waterhouse RM (2015). BUSCO: assessing genome assembly and annotation completeness with single-copy orthologs. Bioinformatics.

[6] Parks DH, Imelfort M, Skennerton CT, Hugenholtz P, Tyson GW (2015). Checkm: assessing the quality of microbial genomes recovered from isolates, single cells, and metagenomes. Genome Res.

[7] Kang DD, Froula J, Egan R, Wang Z (2015). Metabat, an efficient tool for accurately reconstructing single genomes from complex microbial communities. PeerJ.

[8] Brettin T, Davis JJ, Disz T, Edwards R, Gerdes S, Olsen G, Olson R, Overbeek R, Parrello B, Pusch GD, Shukla M, Thomason JA, Stevens R, Vonstein V, Wattam AR, Xia F (2015). Rasttk: a modular and extensible implementation of the rast algorithm for building custom annotation pipelines and annotating batches of genomes. Sci Rep.

[9] Overbeek R, Begley T, Butler RM, Choudhuri JV, Chuang H-Y, Cohoon M, de Crécy-Lagard V, Diaz N, Disz T, Edwards R, Fonstein M, Frank ED, Gerdes S, Glass EM, Goesmann A, Hanson A, Iwata-Reuyl D, Jensen R, Jamshidi N, Krause L, Kubal M, Larsen N, Linke B, McHardy AC, Meyer F, Neuweger H, Olsen G, Olson R, Osterman A, Portnoy V, Pusch GD, Rodionov DA, Rückert C, Steiner J, Stevens R, Thiele I, Vassieva O, Ye Y, Zagnitko O, Vonstein V (2005). The subsystems approach to genome annotation and its use in the project to annotate 1000 genomes. Nucleic Acids Res.

[10] Weisstein EW. CRC Concise Encyclopedia of Mathematics: CRC Press; 2002.

[11] Overbeek R, Olson R, Pusch GD, Olsen G, Davis JJ, Disz T, Edwards R, Gerdes S, Parrello B, Shukla M, Vonstein V, Wattam AR, Xia F, Stevens R (2014). The seed and the rapid annotation of microbial genomes using subsystems technology (rast). Nucleic Acids Res.

[12] Genome Annotation. https://docs.patricbrc.org/tutorial/genome_annotation/annotation.html. Accessed 01 Sept 2019.

[13] Comprehensive Genome Analysis Service. https://docs.patricbrc.org/tutorial/comprehensive-genome-analysis/comprehensive-genome-analysis.html. Accessed 01 Sept 2019.

[14] Using the PATRIC Metagenomic Binning Service. https://docs.patricbrc.org/tutorial/metagenomic_binning/metagenomic_binning.html. Accessed 01 Sept 2019.

[15] Compare Region Viewer. https://docs.patricbrc.org/user_guides/organisms_gene/compare_region_viewer.html. Accessed 01 Sept 2019.

